# Workability among migrant diabetic workers: An occupational health-clinic-based study, Kuwait

**DOI:** 10.1371/journal.pgph.0004911

**Published:** 2025-07-11

**Authors:** Sarah A. Bolbol, Shaikhah M. Al-Fajjam, Al-Anoud E. Al-Ateeq, Shaikha S. Al-Hajeri, Salwa F. El-Saka

**Affiliations:** 1 Occupational Health Administration, Ministry of Health, Kuwait; 2 Department of Community, Environmental and Occupational Medicine, Faculty of Medicine, Zagazig University, Zagazig, Egypt; 3 Department of Public Health and Community Medicine, Faculty of Medicine, Mansoura University, Mansoura, Egypt; Centre of Biomedical Ethics and Culture, PAKISTAN

## Abstract

Diabetes Mellitus (DM) can influence physical and mental health, and workability can be affected by how well workers can manage the disease. So, the aim was to assess workability and identify factors associated with decreased workability among migrant diabetic workers. A cross-sectional study was conducted on 134 migrant diabetic workers in an occupational health clinic in Kuwait. Interview questionnaires on sociodemographic, occupational history, and workability index were used. Workers were subjected to clinical examinations and lab tests including alanine transaminase, aspartate aminotransferase, creatinine, fasting blood sugar, and HbA1C. **The study showed that** among diabetic workers, 19.4% had poor workability, 37.3% had moderate workability, and 34.3% had good to excellent workability. Poor workability was significantly associated with a long history of having DM (≥ 10 years). Workers with poor workability had significantly higher levels of fasting blood sugar (FBS), HbA1c, and creatinine (0.044, 0.019, and <0.001, respectively). Correlation showed that increased level of FBS, HbA1c, and creatinine was significantly associated with decreased levels of workability among diabetic workers (0.04, 0.02, 0.000 respectively). **In conclusion,** although many adults with diabetes remain productive members of the workforce, effective disease management is essential to minimizing complications and reducing its impact on job performance.

## Introduction

Diabetes mellitus (DM) is a chronic disease that occurs when the pancreas fails to produce insulin or when the body cannot effectively use the insulin it generates. It is one of the most prevalent non-communicable diseases that poses a serious threat to public health globally. In 2019, an estimated 463 million people were living with DM, and this number is projected to increase to 578 million by 2030. Of those with DM, three out of four are in the working age group (20–64 years). This corresponds to 351.7 million people and is expected to increase to 417.3 million by 2030. In 2019, the number of deaths among adults aged 20–79 years attributed to DM and its complications was 4.2 million. Additionally, DM is thought to be linked to 11.3% of deaths worldwide from all causes among people in this age group. Around 46.2% of the deaths associated with DM in the working age group occurred in people under the age of 60 years [1].

The Middle East and North Africa (MENA) area had the highest global age-standardized DM prevalence of 12.2% in 2019 among the seven International Diabetes Federation global regions (IDF). Kuwait is one of the countries with the highest diabetes prevalence globally. In 2019, the estimated prevalence of DM among adults in Kuwait was 22.0% [2], which is much higher than the global prevalence of 9.3% [3]. Kuwait’s health system is heavily burdened by the high prevalence of obesity, DM, and related comorbidities in all societal segments [4].

Due to direct or indirect costs like absenteeism and ineffective presence at work, DM imposes a substantial financial burden on society. Effective DM self-management during the workday may be hindered by a heavy workload (limiting the length or frequency of breaks), inadequate job control (unplanned events), and unspoken social norms (discomfort with testing blood glucose or administering insulin) [5].

Diabetes Mellitus will be linked to lower productivity and early retirement among employees. A systematic study in 2013 found that DM reduces the workability (WA) of workers and is likely to exacerbate the burden of DM as the complications become more prevalent in the working-age population. Moreover, a meta-analysis study found that DM was associated with a rapid increase in the risk of disability [6].

The workability index (WAI), a questionnaire created by some Finnish researchers to assess employees’ WA, is a crucial tool that can measure the equilibrium between work needs and employees’ WA in an objective manner. Among the applications of this questionnaire, is the evaluation of the impairment in diabetic workers and to identify workers at risk of work disability [7].

Workability (WA) is the state in which an employee can adjust to the demands of their job, either mentally or physically, depending on their current state of health. People with DM may be less able to work, which leads them to withdraw from work. While some Kuwaiti studies addressed many aspects of diabetes such as quality of life [8], behavior [9], and risk factors among diabetic workers [10], this is the first study, to the best of our knowledge, to assess workability among diabetic workers in Kuwait. We aimed to assess workability and identify factors associated with decreased workability among migrant diabetic workers.

## Subjects and methods

### Ethics statement

Before carrying out the study, approval from The Standing Committee for Coordination of Health and Medical Research was obtained (No#2361/ 2023). Informed written consent was obtained from all participants in the study. They were informed about the aim of the study, and they were reassured about the confidentiality of any obtained information and that the results will be used for research. Workers were informed about their right to reject participation and to withdraw whenever they wanted without giving reasons and with no consequences. This study committed to the Code of Ethics of the World Medical Association (Declaration of Helsinki).

### Study type and setting

A cross-sectional study was carried out over a period of three months from 1^st^ October 2023–31 December 2023 at Shuwaikh Industrial Health Center occupational health clinic, Kuwait.

### Study population

Diabetic workers attending diabetes clinic at Shuwaikh Industrial Health Center monthly. All of them are males, skilled manual workers at auto repair shops working one shift.

### Sampling methods

We used a systematic random sampling technique to select workers who were going to participate in this study. The sample size was calculated using the openepi program, the total number of patients visiting the clinic in a month is 200, using the prevalence of low workability among diabetics from a previous study of 46.6% [11] at confidence interval 95% and power of test 80%, the target sample size was calculated to be 132 diabetic workers as the minimal size calculation therefore, our final sample size was 134 workers. To ensure a random sample, the K interval was calculated to be 2. The first worker was chosen randomly and then every second worker was included till reaching the targeted sample size.

Inclusion criteria: workers agreed to participate by written consent, type II diabetic workers, having DM ≥ 6 months.

Exclusion criteria: workers refused to participate, type I diabetic workers, having DM < 6 months.

### Study tools

1. Questionnaires:

An interview questionnaire was conducted on the patients; the questionnaire was filled by the nurses using a hard copy. Before conducting the study, the questionnaire items were fully explained to the nurses to ensure the accuracy of the collected data.

Questionnaires covered the following:

Questionnaire on sociodemographic and occupational history: data included age, sex, marital status, level of education, history of smoking, duration of work (years of experience), and working hours/day.

Perceived workability evaluated by the Work Ability Index (WAI) questionnaire [12]: Workers were subjected to a standardized questionnaire to assess workability in English and Arabic. English questionnaires were suitably translated into Arabic and back-translated into English by another language expert. A group of experts evaluated the Arabic and English versions for content validity.

The questionnaire consists of 7 parts: (1) Current workability in comparison with the best time of life (scored 0–10). (2) Subjective current workability as regards the physical and mental demands of work (two questions each on a 5-point scale). (3) The number of diseases diagnosed by a physician (scores ranged from 1 = more than 5 diseases to 7 = 0 diseases). (4) Estimated work impairment due to diseases (six-point scale ranging from 1-6). (5) Sick leave in the past 12 months (scores ranged from 5 = 0 days to 1 = 100 days or more). (6) Personal prognosis of workability in the next two years (score of 1 = hardly able to work, 4 = not sure, or 7 = fairly sure). (7) Personal resources in the last few months assessed through three questions (enjoying daily activities, being active and alert, and feeling full of hope about the future). The answers ranged from never = 0 to always = 4. According to the WAI score, workability is categorized into four classes: poor (7–27), moderate (28–36), good (37–43), and excellent (44–49). In the subsequent analyses, both categories good and excellent were combined into one group (good workability).

2. Clinical examinations: included the history of DM, body mass index (BMI), systolic blood pressure (SBP), and diastolic blood pressure (DBP).

BMI classification: underweight: < 18.5, normal weight: 18.5-24.9, overweight: 25-29.9, and obesity: ≥ 30

3. Lab tests: patients in the clinic routinely perform some investigation for follow-up of the disease progress and to determine whether the patients are controlled. These investigations included alanine transaminase (ALT) & aspartate aminotransferase (AST), fasting blood sugar (FBS), and HbA1C.

### Data management

The collected data was entered, checked, and statistically analyzed using the Statistical Package for the Social Sciences (SPSS) program version 29.0. Qualitative variables are expressed as numbers and percentages, and quantitative variables as mean ± S.D. The significance of associations was tested using the ANOVA test (compares several means), and the Chi-square test for categorical variables. Logistic regression analysis both univariate and multivariate was used to detect the significant predictors. Pearson correlation was used to measure the linear correlation between data. The test results were considered significant when the p-value < 0.05.

## Results

The current study was conducted on 134 diabetic workers, 19.4% had poor workability, 37.3% had moderate workability, and 34.3% had good to excellent workability. All workers are males and married. Most workers aged 45 years and older and had a school education. About 60% have been working for 15–30 years. Two-thirds of study participants (67.2%) worked 8 hours or less Table 1. None of the sociodemographic or occupational characteristics were significantly associated with workability Table 2.

**Table 1:** Sociodemographic, occupational, workability characteristics among diabetic workers.

Characteristics	N. (134)	%
**Age (Years)**		
25- < 35	1	0.7
35- < 45	19	14.2
≥45	114	85.1
**Education**		
Illiterate	10	7.5
Read and write	16	11.9
School	93	69.4
High	15	11.2
**Smoking**		
Non-smoker	95	70.9
Current smoker	25	18.7
Former smoker	14	10.4
**Work duration (Year)**		
1- < 15	23	17.2
15- < 30	77	57.5
≥30	34	25.4
**Work Hours**		
≤8 (n.90)	90	67.2
> 8 (n.44)	44	32.8
**Workability classification**		
Poor	26	19.4
Moderate	50	37.3
Good	58	43.3

**Table 2:** Association between workability and sociodemographic and occupational characteristics.

Characteristics	WA N. 134			P-value
	Poor N. (%) 26 (19.4)	Moderate N. (%) 50 (37.3)	Good N. (%) 58 (43.3)	
**Age (Years)**				
25- < 35 (n. 1)	1(100.0)	0 (0.0)	0(0.0)	0.29^†^
35- < 45 (n. 19)	3(15.8)	6(31.6)	10(52.6)	
≥45 (n. 114)	22(19.3)	44(38.6)	48(42.1)	
**Education**				
-Illiterate (n.10)	2 (20.0)	6 (60.0)	2(20.0)	0.07
-Read and write (n.16)	6 (37.5)	6(37.5)	4 (25.0)	
-School (n. 93)	18(19.4)	33(35.5)	42(45.2)	
-High (n.15)	0 (0.0)	5(33.3)	10(66.7)	
**Smoking**				
- Non-smoker (n.95)	18(18.9)	33(34.7)	44(46.3)	0.46
-Current smoker (n.25)	4(28.6)	4(28.6)	6(42.9)	
-Former smoker (n.14)	4(16.0)	13(52.0)	8(32.0)	
**Work duration (Year)**				
- 1- < 15 (n.23)	7(30.4)	7(30.4)	9(39.1)	0.57
- 15- < 30 (n. 77)	14(18.2)	31(40.3)	32(41.6)	
- ≥ 30 (n.34)	5(14.7)	12(35.3)	17(50.0)	
**Work Hours**				
≤8 (n.90)	14(15.6)	36(40.0)	40(44.4)	0.25
> 8 (n.44)	12(27.3)	14(31.8)	18(40.9)	

† Fisher Exact test

Studying the clinical characteristics of diabetic workers, we found a significant association between workability and duration of DM. Workers with a long history of DM showed low workability (45.2%) while (64.1%) of those diagnosed less than five years ago demonstrated good work ability. The presence of additional underlying diseases significantly reduced workability, as a higher number of comorbidities was associated with lower levels of workability (p < 0.001) Table 3.

**Table 3:** Association between workability and clinical characteristics of diabetic workers.

Characteristics	WA (N. 134)			P-value
	Poor N. (%) 26 (19.4)	Moderate N. (%) 50 (37.3)	Good N. (%) 58 (43.3)	
**Duration of DM (Ys)**				
<5 (n.39)	0 (0.0)	14 (35.9)	25(64.1)	<.001^*†^
5- < 10 (n.53)	7(13.2)	23(43.4)	23(43.4)	
≥10 (n.42)	19(45.2)	13(31.0)	10(23.8)	
**Underlying diseases**				
One disease (n.44)	0(0.0)	2(4.5)	42(95.5)	<.001^*†^
Two diseases (n.25)	11(44.0)	1(4.0)	13(52.0)	
Three diseases (n.65)	15(23.1)	47(72.3)	3(4.6)	
SBP (mean±SD)	128.23 ± 21.83	130.62 ± 17.18	134.31 ± 16.66	0.308
DBP (mean±SD)	77.65 ± 13.09	78.84 ± 8.98	79.67 ± 9.19	0.689
**Body Mass Index (BMI)**				
-Underweight (n.1)	0(0.0)	1(100.0)	0 (0.0)	<.001^*†^
-Normal weight (n.32)	0(0.0)	10 (31.3)	22(68.8)	
-Overweight (n.63)	4(6.3)	28(44.4)	31(49.2)	
-Obesity (n.38)	22(57.9)	11(28.9)	5(13.2)	

* Significant <0.05 ^†^ Fisher Exact test.

A logistic regression analysis was conducted to identify significant independent predictors associated with low workability. In the univariate analysis, obesity, a duration of DM more than 10 years, and the presence of more comorbidities were all significantly associated with lower workability. However, in the multivariate logistic regression model, only obesity (OR= 62.8) and DM duration of more than 10 years (OR= 21.3) were the most significant independent predictors of low workability, indicating a strong association even after adjusting for other variables Table 4.

**Table 4:** Sociodemographic, occupational, and clinical risk factors associated with low workability and results of Logistic regression analysis among diabetic workers.

Risk factors	Unadjusted OR (95% CI)^†^	Adjusted OR (95% CI)^‡^
**Age (≥45 Years)**	0.756 (0.266-2.148)	1.263 (0.149-10.675)
**Education (Illiterate)**	0.597 (0.340-1.051)	0.615 (0.192-1.969)
**Smoking (Current smoker)**	0.968 (0.560-1.675)	0.402 (0.135-1.201)
**Work duration (≥30 Years)**	0.622 (0.317-1.218)	0.386 (0.104-1.442)
**Work Hours (>8 hrs)**	2.036 (0.849-4.882)	1.910 (0.424-8.597)
**Duration of diabetes (≥10 yrs)**	7.258 (3.052-17.260)^*^	21.320 (3.847-118.167)^*^
**Underlying diseases (Three diseases)**	0.468 (0.262-0.835)^*^	0.381 (0.110-1.321)
**SBP (mean±SD)**	0.968 (0.961-1.011)	0.991 (0.934-1.052)
**DBP (mean±SD)**	0.984 (0.942-1.027)	0.927 (0.871-1.084)
**Body Mass Index (Obesity)**	21.586 (6.774-68.784)^*^	62.809 (6.732-586.000)^*^

† Univariate Logistic regression ^‡^ Multivariate Logistic regression ^*^Significant <0.05.

OR= odds ratio.

Lab investigations among diabetic workers showed that workers with poor workability had significantly higher levels of FBS, HbA1c, and creatinine (0.044, 0.019, and <0.001, respectively) Table 5.

**Table 5:** Association between workability and laboratory findings of diabetic workers.

Characteristics	WA			P-value
	Poor Mean±SD	Moderate Mean±SD	Good Mean±SD	
**FBS**	10.45 ± 3.71	9.14 ± 3.29	8.5 ± 3.01	0.044^*^
**HbA1C**	9.19 ± 1.68	8.26 ± 1.80	8.07 ± 1.60	0.019^*^
**Creatinine**	104.15 ± 19.45	85.16 ± 19.36	84.46 ± 20.03	<0.001^**^
**Cholesterol**	3.95 ± 1.14	4.31 ± 1.12	4.16 ± 0.88	0.358
**TG**	2.03 ± 1.03	1.82 ± 1.07	1.73 ± 0.88	0.448
**ALT**	29.96 ± 12.39	34.88 ± 16.76	37.28 ± 20.60	0.225
**AST**	26.87 ± 10.74	26.00 ± 9.22	30.01 ± 15.32	0.227

* Significant <0.05 ^**^Highly significant <0.01.

Studying the correlation between workability and some laboratory findings, we found that increased levels of FBS, HbA1c, and creatinine was significantly associated with decreased workability among diabetic workers Table 6.

**Table 6:** Correlation between workability and laboratory findings of diabetic workers.

Variables (Means ± SD)	Workability mean± SD:34.61 ± 8.79
	r (p)
**FBS (9.12 ± 3.31)**	-0.17(0.04)^*^
**HbA1c (8.35 ± 1.73)**	-0.18 (0.02)^*^
**Creatinine (88.54 ± 20.98)**	-0.25 (0.00)^**^

* Significant <0.05 ^**^Highly significant <0.01.

## Discussion

Kuwait ranks among the top twenty countries globally with the highest rates of diabetes (DM) prevalence in adults [13]. In Kuwaitis, the prevalence of diabetes and prediabetes was 21.8% and 11.1%, respectively, while the prevalence in non-Kuwaitis was 18.2% for diabetes and 14.3% for prediabetes [3]. This study aimed to identify factors associated with decreased workability among migrant diabetic workers.

Diabetes mellitus negatively impacts workers’ occupational lives. Based on the international classification of functioning disability and health, Kazemi et al. [14] assessed the performance of 94 diabetic patients in Iran. They found that workability in patients with DM is not at the desired level, and their workability decreased as their fasting blood sugar increased. Alsalem and Alhaiz [1] Conducted a systematic review of multiple studies and concluded that diabetes mellitus (DM) and its complications had a negative impact on employee performance and overall productivity. While these studies provide a broad insight into the burden of DM on the workplace, the results may vary depending on the country where the study was performed.

In the current study, more than half of the sample (56.7%) had poor to moderate workability. This finding was consistent with the study of Elhadidy et al. [15] and Motamed et al. [11] who showed that the majority of their sample had poor to moderate workability (74.6% and 75%, respectively). While Mc Carthy et al. [16], demonstrated a lower percentage (26%) in their study. This variation in perceived workability could be attributed to differences in workplace support (employers and partners), insurance organizations, and access to health services [7]. The present study revealed that the presence of a long history of DM, more diseases accompanying DM, and high BMI were significantly associated with poor workability. This result is supported by other studies which found a significant association between DM comorbidities and work disability in diabetic workers [11,17,18], and the study of Andersen et al. [19] who found that BMIs above the normal range are progressively associated with lower workability.

Regression analysis showed that obesity and DM duration of more than 10 years were the most significant independent predictors of low workability. These findings are unsurprising, considering the migrant status of the workers, living away from their families, and they often rely on inexpensive, high-carbohydrates, low-nutritional-value foods due to limited resources and time constraints. Furthermore, long duration of having diabetes frequently linked to the development of complications that impair physical functioning and reduce the workers’ capacity to carry out their job responsibilities effectively.

The present data showed that workers with poor workability had significantly higher levels of FBS and HbA1c. In consistency with this finding, a Finnish study by Hakkarainen et al. [20] showed that high HbA1c values, which indicate inadequate glycemic control, were linked to decreased workability in people with diabetes. Moreover, an Iranian study demonstrated a significant difference between the mean of FBS and HbA1C in terms of workability in patients with DM [11].

Migrant workers tend to have unhealthy lifestyles. They are usually employed in “3D jobs” which refers to dirty, dangerous, and difficult [21]. The majority of migrant workers occupy positions that do not match the professional profile of the immigrant. They usually work longer hours in risky jobs when compared to local workers [21]. Furthermore, Alahmad et al. [22] considered migrant workers as a neglected vulnerable group. Lack of access to healthy food with a low glycaemic index and depending on less nutritive caloric fast meals during their long work hours makes it difficult to maintain appropriate blood sugar.

Persistent high blood sugar levels in DM are accompanied by long-term damage, impaired function, and eventual failure of various organs, particularly the eyes, kidneys, nerves, heart, and blood vessels [23]. The current findings revealed that individuals with low workability had notably elevated creatinine levels. This result is consistent with the study by Motamed et al. [11], which found significantly higher mean levels of creatinine and blood urea nitrogen (BUN) in diabetic workers with low workability. Also, Alkathiri et al. [24] highlighted the critical relation between poor glycaemic control and impaired function of vital organs, such as the kidney.

Kuwait has frequently experienced hourly temperatures above 50°C and daily highs exceeding 40°C in recent years. Hotter summers threaten workers’ health [25]. The state of dehydration caused by working in hot weather for long hours adds to the burden of DM on kidney function and consequently, this affects work performance.

According to the recommendations of consensus treatment panels, most patients failed to meet the targets for reasonable HbA1C. Regional estimates of North Africa and the Middle East reported that only 37% of the patients with type 2 diabetes had HbA1C < 7.0% in 2021 [26].

Several studies investigated the association of DM and cognitive dysfunction they agreed that inadequate control of diabetes mellitus (DM) contributes to a higher likelihood of cognitive dysfunction, emphasizing the need for strict glycemic regulation to minimize this risk [27–29]. At work, especially if mentally demanding, cognitive functions, such as memory, attention, problem-solving, decision-making, and processing speed, are crucial for performance in most tasks. Therefore, maintaining cognitive functions by controlling DM helps to improve workability.

Furthermore, results of the current study showed that increased level of FBS, HbA1c, and creatinine was significantly associated with decreased levels of workability among diabetic workers. Similarly, a study by Wali et al. [30] in Egypt revealed a significant negative correlation between the performance of diabetic workers and FBS glycated Hb and creatinine levels.

This study was the first to evaluate the workability of diabetic workers in Kuwait. Nevertheless, it had some limitations as the cross-section design together with the absence of control group made it difficult to establish a causal relationship. The selected group of manual workers tends to have uncomplicated controlled DM compared to other non-manual workers. Additionally, self-reported data may affect the accuracy of the responses to some of the questions as migrant workers tend to reveal a high level of workability to maintain their livelihood.

## Conclusion

More than half of the participants demonstrated weak to moderate levels of workability. Based on the study findings, the implementation of health and safety policies for diabetic workers to allow regular healthy meals and snack breaks are strongly recommended. Moreover, encouraging routine checks for blood glucose levels, allowing flexible work hours, and conducting targeted DM screening and awareness campaigns for migrant workers are effective interventions to help them better manage their health condition. This, in turn, would improve their workability.
